# Psychological Factors Influencing Attitudes towards Euthanasia, Assisted Suicide and Palliative Care among Medical Students and Doctors in Training

**DOI:** 10.3390/healthcare12080833

**Published:** 2024-04-15

**Authors:** Maria Forycka, Magdalena Liberacka-Dwojak, Wojciech Leppert, Paweł Suchecki, Natalia Suchecka, Bartłomiej Ast

**Affiliations:** 1 Department of Palliative Medicine, Institute of Medical Sciences, Collegium Medicum, University of Zielona Góra, ul. Zyty 28, 65-046 Zielona Góra, Poland; m.forycka@inm.uz.zgora.pl; 2 Department of Psychology, Kazimierz Wielki University, ul. Leopolda Staffa 1, 85-867 Bydgoszcz, Poland; mldwojak@ukw.edu.pl; 3 University Hospital in Poznań, Osiedla Rusa 55, 61-245 Poznań, Poland; 4 MediCenter Primary Care Physicians, ul. 3 Maja 13, 99-400 Łowicz, Poland; suchecki.kg@gmail.com (P.S.); n.nataliasuchecka@gmail.com (N.S.); 5 Department of Care and Treatment Facility, Józef Struś Multispecialist Municipal Hospital, ul. Szwajcarska 3, 61-285 Poznań, Poland; bartek.ast@poczta.onet.pl

**Keywords:** euthanasia, trainee physicians, palliative care, medical students, Polish and classical philology students

## Abstract

**Background:** This study aimed to analyse the attitudes of medical students, Polish and classical philology students and trainee doctors towards the legalisation and practice of euthanasia and assisted suicide, to explore their beliefs about palliative care and to identify the cognitive, behavioural and emotional factors influencing these attitudes. **Methods:** An anonymous 22-question survey was sent by email to 670 participants, who comprised students of medicine, students of Polish and classical philology and trainee physicians. **Results:** Out of the 670 people invited to the survey, 313 (46.72%) responded; 215 (68.69%) and 112 (35.80%) participants supported the legalisation of euthanasia and assisted suicide, respectively. No differences were found between the respondent groups studied. The respondents’ attitudes were influenced by religion, place of residence and professed values in the doctor–patient relationship. Among the medical students and trainee doctors surveyed, the declared willingness to perform euthanasia was lower, with 90 (43.7%) people, than the support for its legalisation, with 135 (65.5%) people. Significantly higher support for palliative care was expressed by fifth- and sixth-year medical students and trainee doctors, with 88 respondents (89.89%), less support was expressed by first- and fourth-year medical students, with 74 respondents (68.5%), and the lowest support was observed among Polish and classical philology students, with 63 respondents (58.9%). **Conclusions:** The legalisation of euthanasia and assisted suicide was supported by more than two-thirds and one-third of all the respondents, respectively, with the majority of medical students and trainee doctors surveyed expressing uncertainty or lack of readiness towards their practice. More than 70% of all the respondents showed a positive opinion towards palliative care, with the lowest support being among Polish and classical philology students.

## 1. Introduction

According to European Association for Palliative Care (EAPC)**,** euthanasia is defined as a doctor intentionally killing a person by the administration of drugs at that person’s voluntary and competent request, and physician-assisted suicide is defined as a doctor intentionally helping a person to commit suicide by providing drugs for self-administration at that person’s voluntary and competent request [1,2]. In Polish law, euthanasia and assisted suicide (article 150, point 1 and 2 of the Penalty Code) are forbidden. The penalty for both offences ranges from 3 months to 5 years of imprisonment. However, euthanasia is here defined as an action at the request of and as an act of mercy for the victim; therefore, in special circumstances, a court may give a milder sentence or grant complete exemption from punishment [3].

Many countries, often as a result of social and political pressure, have allowed their citizens full or partial legal access to euthanasia and assisted suicide. In addition, euthanasia and physician-assisted suicide face opposition from many quarters, including doctors, nurses and other healthcare professionals, especially those involved in palliative care, geriatrics and psychiatry, and they are also prohibited by law in some countries [4,5,6].

The imperative of happiness has removed illness and suffering as inseparable elements of life from public discourse and private space [7], and death was to become purely “technical”, quick and discreet [8]. Meanwhile, the number of patients with chronic, progressive diseases with an unfavourable prognosis is increasing, often resulting in fears of losing control over their own bodies and dependence on others as well as fears of disability and suffering [9,10]. In a context of radical and rapid social change and ideological differences, it seems reasonable to encourage the public to reflect more deeply on dying with respect for the dignity of patients and their relatives, taking into account the important role of palliative care, which can provide comprehensive and multidimensional care to patients and their relatives until the end of the patient’s life and to relatives also after the patient’s passing.

The results of studies regarding medical students’ attitudes toward euthanasia and assisted suicide suggest significant discrepancies, e.g., euthanasia and assisted suicide have proponents amounting to 24–97.4% and 13–69%, respectively [9,10,11,12,13,14,15,16]. There is more support for euthanasia in countries where it is legal, e.g., in Belgium 97.4% [17] and in Canada 88% [18], compared to countries where it is forbidden, e.g., in China 41.2% [15] and Germany 19.2% [16]. Factors that significantly impact negative attitudes toward euthanasia and assisted suicide comprise religion [11,19], a higher year of study [20,21], a lack of law acceptance and a concern for abuse [22]. Factors that significantly impact the support for euthanasia or assisted suicide comprise patient autonomy and the provision of suffering relief [11], and irrelevant variables comprise age and socio-economic status [11,23,24]. Studies comparing medical and other fields of study unveiled that medical students demonstrated less support for euthanasia and assisted suicide compared to students of other fields. The only exception was law students, whose attitudes toward euthanasia were similar to those of medical students [12,25].

The aim of this study was to compare attitudes toward euthanasia, assisted suicide and palliative care among medical students, trainee doctors and Polish and classical philology students (philology—part of humanistic sciences combining the study of the history of language, linguistics and the historical study of literary texts), taking into account factors such as age, religion, place of residence and year of study. The secondary aims included assessing the impact of cognitive, behavioural and emotional factors on these attitudes. It was assumed that knowledge of definitions (cognitive component), the potential possibility of intervention and decision (behavioural component), values and own experience (emotional component) may influence these attitudes of respondents.

It was hypothesised that medical students and doctors in training would show less support for conducting (the practice of) euthanasia and assisted suicide compared to Polish and classical philology students. The supportive questions are as follows:

1. Does more knowledge about the object of attitude (euthanasia, assisted suicide and palliative care) impact these attitudes?
2. Does the perspective of potentially deciding about euthanasia or potentially committing euthanasia or assisted suicide impact these attitudes?
3. Do experience of own disease or disease of a close person or value system have an impact on these attitudes?

## 2. Material and Methods

**Figure 1:**
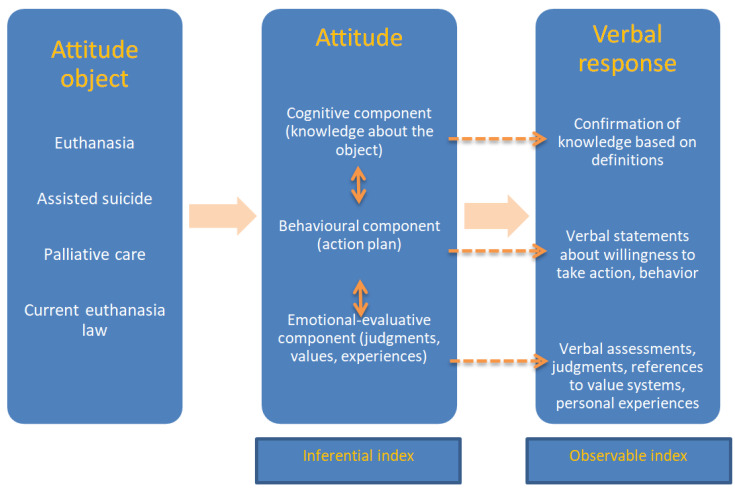
Conceptualization of the relationship between attitude object, attitude observed and verbal response (own source).

The survey of respondents’ attitudes was based on structural theory. An individual’s attitude towards some tangible or abstract object is a relatively permanent evaluation that values that object positively or negatively [26], which plays a mediating role between stimulus and response [27] and helps to predict and understand human behaviour [28]. Attitudes are complex structures from which many elements can be extracted. Three core components of attitude have been identified: a cognitive component, a behavioural component and an emotional component [29,30,31]. The existence or absence of particular components and the relationships between them can be useful in understanding more fully human attitudes, including the behaviour of individuals and social groups. The components of attitudes are difficult to observe and require an appropriate empirical approach. Individual responses to questions (verbal reactions) with cognitive, behavioural and emotional load were treated as observable consequences of hypothetical (inferential) attitude indicators (Figure 1). Indeed, it can be assumed that the key determinant of surveyed doctors’ and students’ behaviour will be their attitudes towards given practice, the latent form of which will be revealed through the behavioural, emotional and cognitive components observable in verbal responses.

This study used a proprietary questionnaire with 22 questions, including 18 closed questions and 4 supplementary questions to provide justification for the answers that were given (Supplementary Material File S1). The choice of questions was based on an analysis of the literature [11,29,32,33] and the results of our own studies [23,24]. Before starting the study, a linguistic evaluation and an evaluation of the understanding of the content of the questionnaire was conducted by two independent experts in linguistics. This questionnaire comprised questions regarding the socio-demographic data of the respondents, their attitudes toward euthanasia, assisted suicide and palliative care and current Polish law in this regard. The questionnaire was based on a conceptual framework relating to the observed verbal responses pertaining to the cognitive, emotional and behavioural components of the subjects’ attitudes. This questionnaire was sent to all medical students of all years of study and all trainee doctors who completed their studies at one academic year of the same Collegium Medicum of the University of Zielona Góra and all Polish and classical philology students of all years of study of Adam Mickiewicz University in Poznań, Poland. The respondents received an email with an information about the topic of the study with a request to provide informed consent for completely voluntary participation in the study. A link to the questionnaire was enclosed. As the study did not concern patients nor it was considered a medical experiment, the procedure of informed consent was limited to filling in the questionnaire, which was deemed as agreement to participate in this study. The study protocol was approved by the Bioethics Committee (nr KB–UZ/30/2021). The study data are available upon request from the authors of the study.

The subjects were divided into 3 groups. Group 1 included first- to fourth-year medical students who had not taken a course in palliative medicine. Group 2 comprised fifth- and sixth-year medical students and trainee doctors who had taken the compulsory fifth-year palliative medicine course. Group 3 included first- to fifth-year Polish and classical philology students who had not taken a course in palliative medicine.

The results were analysed by attitudes towards euthanasia, attitudes towards assisted suicide and attitudes towards palliative care. Questions relating to familiarity with the definitions of euthanasia, assisted suicide, palliative care and persistent therapy were related to the cognitive component of attitudes (questions 1, 5, 7 and 8). To observe the behavioural component, medical students and doctors in training were asked about their willingness to carry out euthanasia and assisted suicide (questions 9 and 10). All respondents were asked a question about undergoing euthanasia or assisted suicide in the event of their own terminal illness (question 17). Questions relating to the experience of their own illness, illness of a loved one, the most important value in the patient–doctor relationship and assumed difficulties in caring for a patient with advanced disease were asked to measure the emotional and evaluative components of attitudes (questions 16, 18, 19, 20, 21 and 22). Descriptive statistics of the study variables are presented. A chi-squared test was used to compare demographics, question responses and differences between respondents; a *p*-value < 0.05 was considered significant.

## 3. Results

Of the 670 invited, 313 responded to the survey (response rate 46.72%). Group 1 comprised 108 (34.5%) first- to fourth-year medical students, group 2 comprised 98 (31.31%) respondents, including 86 fifth- and sixth-year medical students and 12 doctors in training and group 3 comprised 107 (34.19%) first- to fifth-year students of Polish and classical philology. The majority of the participants in this study were female 223 (71.2%). The age of all subjects was 23.52 ± 3.26 (range 18–43 years); in groups 1, 2 and 3, the ages were 22.49 ± 2.56, 25.6 ± 1.7 and 22.64 ± 4.02 years, respectively. In terms of religion, Roman Catholic affiliation, atheism and agnosticism were declared by 169 (54%), 96 (30.68%) and 20 (6.39%) respondents, respectively. The sociodemographic characteristics of the respondents are shown in Table 1.

### 3.1. Attitudes towards Euthanasia and Assisted Suicide and Palliative Care and Sociodemographic Differences

Of all the respondents surveyed, 215 (68.69%) were in favour of the legalisation of euthanasia, and 112 (35.80%) were in favour of the legalisation of assisted suicide; there were no differences between the respondents from the surveyed groups in this regard, either in relation to the legalisation of euthanasia, *p* = 0.072, or in relation to the legalisation of assisted suicide, *p* = 0.398. A large majority, 225 (71.88%) of the respondents, considered palliative care as providing a dignified life (Table 2).

Attitudes towards euthanasia differed by (*p* = 0.037) place of residence. Respondents living in rural areas, 12 (20%) people, and in cities with from 51,000 to 100,000 inhabitants, 9 (36%) people, were more likely to be unsure as to whether euthanasia should be legalised compared to those living in cities with more than 200,000 inhabitants, 3 (12.5%) people. Religion differentiated (*p* < 0.001) attitudes towards euthanasia and assisted suicide. The legalisation of euthanasia was supported by 88 (91.7%) and 89 (52.7%) people, and that of assisted suicide was supported by 48 (50%) and 40 (23.7%) people, among atheists and Catholics, respectively, in both cases.

Attitudes towards palliative care were differentiated by religion (*p* = 0.008), field of study (*p* < 0.001) and year of study (*p* = 0.012). Among Catholics and agnostics, more people, 132 (78.1%) and 17 (85%), respectively, compared to atheists, 62 (64.6%), claimed that palliative care provides patients with a dignified life. More support for palliative care was expressed by fifth- and sixth-year medical students and doctors in training, with 88 (89.89%) people (group 2), with less support, with 63 (58.9%) people, among students of Polish and classical philology (group 3).

### 3.2. Cognitive Component: Respondents’ Knowledge of Attitude Objects

Knowledge of the definition of euthanasia was declared by 299 (95.5%) people, assisted suicide by 181 (57.8%) people and palliative care by 274 (87.5%) people (Table 3). Knowledge of the definition differentiated (*p* < 0.001) attitudes towards assisted suicide. Of the 181 people who declared knowledge of the definition of assisted suicide, 86 (47.5%) were in favour of its legalisation, 45 (24.9%) were against it and 50 (27.6%) were undecided. Of the 132 people who declared being unfamiliar with the definition of assisted suicide, 26 (19.7%) people supported its legalisation, 31 (23.5%) people were of the opposite opinion and 75 (56.8%) respondents were undecided.

### 3.3. Behavioural Component: Readiness to Euthanise

The question on willingness to perform euthanasia and assisted suicide, if legalised, was asked to medical students and doctors in training (groups 1 and 2, *n* = 206). Willingness to perform euthanasia and assisted suicide on a patient was declared by 90 (43.7%) and 53 (25.7%) people, respectively, a lack thereof was expressed by 36 (17.5%) and 47 (22.8%) people, respectively, and an attitude of uncertainty was expressed by 80 (38.8%) and 106 (51.5%) people, respectively (Table 4).

The respondents’ declared willingness to perform euthanasia differentiated (*p* < 0.001) their attitudes towards the legalisation of euthanasia and assisted suicide and their attitudes towards palliative care. Respondents declaring willingness to euthanise were more likely to be in favour of legalising euthanasia and assisted suicide, with 85 (94.4%) and 42 (46.7%) respondents, respectively, compared to respondents declaring a lack of willingness to euthanise, with 4 (11.1%) and 3 (8.3%) respondents, respectively. Respondents who declared that they were not ready to perform euthanasia were mostly (*p* < 0.001) against the legalisation of euthanasia and assisted suicide, with 24 (66.7%) and 23 (63.9%) persons, respectively, compared to those declaring that they were ready to perform euthanasia, with 1 (1.1%) and 13 (14.4%) persons, respectively. Fewer (*p* = 0.015) respondents declaring willingness to euthanise said that palliative care provides patients with a dignified life, with 63 (70%) persons, compared to respondents declaring no willingness to euthanise, with 34 (94.4%) persons.

The declared willingness of medical students and doctors in training to assist a patient’s suicide differentiated their attitudes towards the legalisation of euthanasia (*p* < 0.001), assisted suicide (*p* < 0.001) and attitudes towards palliative care (*p* = 0.015). Participants who expressed a willingness to assist in the suicide of a patient were most often (*p* < 0.001) in favour of legalising euthanasia and assisted suicide, with 42 (46.7%) and 45 (84.9%) respondents, respectively. Respondents who did not express readiness to assist in the suicide of a patient were more often (*p* < 0.001) opposed to the legalisation of euthanasia and assisted suicide, with 23 (63.9%) persons and 34 (72.3%) persons, respectively. An undecided attitude towards the legalisation of euthanasia and assisted suicide was expressed by the hesitant respondents, with 39 (48.8%) and 68 (64.2%) persons, respectively. The majority, 162 (78.6%), of the medical students and doctors in training, regardless of their willingness to perform euthanasia and assisted suicide, stated that palliative care provides patients with a dignified life.

### 3.4. Behavioural Component: Willingness to Undergo Euthanasia in Case of One’s Own Illness

The question regarding undergoing euthanasia or assisted suicide, in the event of one’s own advanced illness, was asked to all respondents, of whom euthanasia and natural death would be chosen by 134 (42.8%) and 130 (41.5%) people, respectively. The majority of respondents who declared that they would choose euthanasia in case of their own advanced illness supported the legalisation of euthanasia, with 124 (95.5%) people, and assisted suicide, with 53 (39.6%) people (Table 4). No one was against the legalisation of euthanasia, 24 (17.9%) people were against the legalisation of assisted suicide and 10 (7.5%) and 57 (42.5%) people were undecided about the legalisation of euthanasia and assisted suicide, respectively. Within this group, 89 (68.4%) people claimed that palliative care provides a dignified end of life. For the choice of assisted suicide, in case of one’s own advanced illness, the legalisation of euthanasia, assisted suicide and the claim that palliative care provides a dignified life were supported by 24 (80%), 25 (83.3%) and 22 (73.3%) people, respectively. Of the respondents who would choose natural death in the event of their own advanced illness, 53 (40.8%), 57 (43.8%) and 104 (80%) were in favour of legalising euthanasia and assisted suicide and supported the claim that palliative care provides a dignified end of life, respectively.

### 3.5. Emotional Component: Experiences, Values and Judgements Influencing Attitudes

Of all the respondents, 169 (54%) had no personal experience of caring for a patient with advanced disease, 185 (59%) had not accompanied the death of a loved one and 263 (84%) had not experienced their own chronic illness. Those who were not diagnosed with a chronic disease were more likely (*p* = 0.038) to support the legalisation of assisted suicide, with 98 (37.3%) people, compared to the respondents who had a chronic disease, with 14 (28%) people.

According to 155 (49.5%) people, the most important value in the doctor–patient relationship was the absolute freedom of choice of the patient, with 88 (28.1%) people choosing ethical norms and respect for the patient’s life and 70 (22.4%) people choosing other values without stating which value was meant despite being able to specify it (Table 5). Attitudes towards euthanasia and assisted suicide differed according to the selected most important value in the doctor–patient relationship. Support for the legalisation of euthanasia and assisted suicide was higher (*p* < 0.001) among respondents according to whom the most important value in the doctor–patient relationship was absolute freedom of choice for the patient, with 143 (92.3%) and 45 (64.3%) persons, respectively, and the other most important value in the doctor–patient relationship was shared by 74 (47.7%) and 27 (38.6%) persons, respectively, compared to those who believed that the patient’s life should always be respected, even at an advanced stage of an incurable disease, with 27 (30.7%) and 11 (12.5%) persons, respectively. Respondents for whom ethical norms were the most important value in the doctor–patient relationship were more likely (*p* < 0.001) to be against the legalisation of euthanasia and assisted suicide, with 35 (39.8%) and 39 (44.3%) respectively, than those preferring absolute freedom of choice, with 3 (1.9%) and 22 (14.2%) respectively, and those indicating other values, with 8 (11.4%) and 15 (21.4%), respectively. Indecision towards the legalisation of euthanasia and assisted suicide was more frequently (*p* < 0.001) demonstrated by those for whom ethical norms were most important in the doctor–patient relationship, with 26 (29.5%) and 17 (24.3%) persons, respectively, followed by those with other values, with 38 (43.2%) and 28 (40%) persons respectively, compared to respondents for whom absolute freedom of choice for the patient mattered most, with 59 (38.1%) and 9 (5.8%) persons, respectively.

## 4. Discussion

A questionnaire survey was carried out on attitudes towards euthanasia, assisted suicide and palliative care observed among 107 students of Polish and classical philology, 108 students in the first–fourth year of medicine, 86 students in the fifth and sixth year of medicine and 12 doctors in training. The majority of the respondents were in favour of the legalisation of euthanasia, whereas in terms of attitudes with regard to the legalisation of assisted suicide, the majority were undecided. The majority of the respondents showed a positive attitude towards palliative care, with there being greater support for palliative care among medical students in their fifth and sixth year of study and among doctors in training, i.e., the respondents who had taken the compulsory 30-h course in palliative medicine. No differences were observed between students of different faculties and attitudes towards euthanasia. 

The majority of the respondents claimed to have knowledge of attitude objects. In the case of having less knowledge regarding assisted suicide, a less decisive attitude towards this procedure was observed in most students. The results regarding the students’ attitudes toward assisted suicide were divergent. In some studies, more students supported the legalisation of assisted suicide than that of euthanasia [16,34], and there are other studies in which less students supported assisted suicide than euthanasia [15,18]. In some countries, an indirect way of introducing euthanasia is a decriminalisation of assisted suicide [35]. From an ethical point of view, physicians seem to prefer the practice of assisted suicide because it has less transgressive meaning compared to euthanasia; in the case of assisted suicide, a patient takes a lethal dose of a drug, and thus the moral burden put on the physician seems to be lower [36]. In our study, a significant disparity was observed between the knowledge of the definition of euthanasia held by 299 (95.5%) of all the respondents, including 196 (95.14%) medical students and doctors in training, and the knowledge of the definition of assisted suicide held by 181 (57.8%) of all the respondents and 132 (64.07%) of the medical students and doctors in training. This may be an explanation of less support for the practice of assisted suicide. Although this discrepancy does not directly affect students’ attitudes, it does indicate that knowledge regarding the differentiation of procedures hastening death has little presence in the public consciousness and is mainly limited to euthanasia.

An examination of the behavioural component inferred from the reported willingness to perform euthanasia and assisted suicide and to undergo them in the event of one’s own advanced illness revealed a relationship between the potential for a specific action and attitude. The vast majority of the students and doctors in training willing to perform euthanasia and assisted suicide declared support for the legalisation of these practices. Similarly, support for the legalisation of euthanasia and assisted suicide was higher among the respondents who would choose the above procedures in the event of their own advanced illness. 

Declared religious affiliation differentiated the students’ attitudes towards euthanasia. Those who declared a belief in atheism were the most supportive of euthanasia, while Roman Catholic students were the most opposed to it. Euthanasia was supported by 40 (23.7%) students declaring Roman Catholic affiliation, but there was a significant percentage of 70 (41.4%) being undecided in this group. Religious affiliation is one of the most consistent arguments against euthanasia, regardless of religion, as most religions treat life and suffering as a temporal human condition and death as a natural transition to some other better existence [37,38,39].

Support for the legalisation of euthanasia was declared by 135 (65.5%) medical students and doctors in training, of whom 90 (66.66%) would be prepared to perform it. The above may suggest that the respondents would exempt themselves from the right to interfere in the decisions of individuals, including social decisions, without forcing everyone to make choices according to their own ethical and moral convictions, assuming respect for their freedom of choice and moral and ethical principles. The question arises, however, in addition to a number of important ethical issues, as to what extent will the legislator guarantee the freedom of a doctor to refuse procedures that hasten death and thus ensure the independence of his or her practice. At the same time, there is the question of how will patients and physicians be assured of the freedom of choice with respect to a potential use of persistent therapy that is associated with medical procedures that may prolong patients’ dying and intensify patients’ suffering.

Studies conducted to date have highlighted that medical students are more reluctant to euthanise compared to students in other disciplines and the general public [24,39,40,41]. In the study group, support for euthanasia was found to decrease with increasing years of study [21]. Trends in the opposite direction were observed in students’ attitudes towards the current law, with the majority of upper-year students considering it too restrictive. The most decisive opinion that the current euthanasia legislation in Poland is too restrictive was shown by fifth- and sixth-year medical students and doctors in training.

In Poland, there has been an increase in support for legalising euthanasia among medical students compared to previous years; in 2009, medical students were 34% in favour of legalising euthanasia, and 49% were against it, (*n* = 263) [42]; in 2013 (*n* = 588), almost 30% were in favour, and over 47% were against it [18]. Similarly, increasing support for the legalisation of euthanasia and assisted suicide among medical students has been observed in other countries [43]. However, in the last decade, analyses of medical students’ attitudes have shown significant differences in opinion regarding support for the legalisation of euthanasia, ranging from 19–65% [16,40,44,45,46], with support for the legalisation of euthanasia and assisted suicide being greater in western European countries where they have been legalised [47]. The increase in support for laws accepting euthanasia and assisted suicide in Europe and on other continents contributes to public interest in the solutions that individual countries offer to patients with advanced illness as well as to stronger opinions regarding accepting or rejecting these practices. In Poland, palliative care is strongly integrated with the healthcare system [48,49]; 522 medical entities in Poland provide services in the field of palliative and hospice care, of which 450 (84.4%) are exclusively for adults, 26 (4.9%) are exclusively for children and 54 (10.1%) are for both adults and children [50]. Theoretical and practical classes in palliative care are obligatory for the curriculum of nursing studies at a Bachelor’s degree level. The teaching of palliative medicine for medical students depends on the decisions of authorities of a given medical university or collegium medicum of a university. However, in the majority of medical universities and collegia medica of universities, palliative care is being taught. The positive attitudes of all the surveyed respondents toward palliative care indicate that the intensive development of palliative care translates into increased societal awareness and acceptance of this form of medical care.

The vast majority of the medical students, doctors in training and students of Polish and classical philology in this study considered the absolute freedom of choice of the patient to be the highest value in the doctor–patient relationship. Such a declaration suggests the concern of the surveyed students regarding patient autonomy and rights. It can be assumed that this choice is based on an attitude of empathy, sometimes identified with a projection of the assumed own suffering and choice in a similar situation. According to other studies, this type of reaction is intuitive and often automatic and is not based on reasoned reflection [26], and it may also be related to the social tendency to affirm individual freedom as a fundamental value [51].

The value of individual freedom undoubtedly influenced the respondents’ support for euthanasia and assisted suicide, but this factor did not have a significant impact on the students’ attitudes towards palliative care. The strong support of the medical students and doctors in training for palliative care indicates that the respondents considered this form of care of patients as legitimate and effective. From the consistent positive attitude towards palliative care, it can be inferred that the medical students and doctors in training surveyed considered assisted forms of ending life (physician-assisted dying) as definitive and alternative to palliative care.

The limitations of the study include the undersized group of dual-degree students from two universities in one country and the small number of doctors in training surveyed. The study used a self-administered questionnaire that was not subjected to a psychometric evaluation. The attitudes studied relate to hypothetical situations that do not necessarily reflect the choices made in a real-life situation concerning patients and oneself.

## 5. Conclusions

Despite its limitations, this is the first study to examine attitudes towards euthanasia, assisted suicide and palliative care among students of all years of medicine, doctors in training and students of all years of Polish and classical philology. An attempt was also made to analyse the consistency of attitudes by isolating cognitive, behavioural and emotional determinants. The majority of the respondents were in favour of the legalisation of euthanasia but were undecided regarding the legalisation of assisted suicide. This points to a further need of education in the ethical and legal aspects of end-of-life care in order to prepare future physicians for decisions associated with palliative care provision for patients and families. In our study, it was confirmed that the declared religious affiliation of respondents was the most important argument against euthanasia. The relatively unchangeable impact of religious beliefs on medical students’ and trainee doctors’ attitude toward euthanasia and assisted suicide raises a question to what extent may it impact other practices and clinical decisions made. This might be an interesting area of the future research. There was a decrease in support for euthanasia with year of study, with a concurrent declaration of the medical students and doctors in training that the absolute freedom of the patient’s choice was the most important value in the relationship between patient and physician also being observed. The strong support from medical students and trainee doctors for palliative care indicates an increase in awareness and trust for this type of care. It also suggests an importance of the introduction of palliative medicine into curricula of clinical classes for medical students. 

## Figures and Tables

**Table 1:** Sociodemographic characteristics.

		Total	Degree Course		
			First- to Fourth-Year Medical Students	Fifth- to Sixth-Year Medical Students and Doctors in Training	First- to Fifth-Year Students of Polish and Classical Philology
		(*n* = 313)	(*n* = 108)	(*n* = 98)	(*n* = 107)
Number of students (%)		100%	34.5%	31.31%	34.19%
Age	(mean ± SD)	23.52 ± 3.26	22.49 ± 2.56	25.6 ± 1.7	22.64 ± 4.02
	Range	18–43	19–33	23–33	18–43
Gender	Men	27.2%	30.6%	41.8%	10.3%
	Women	71.2%	68.5%	57.1%	86.9%
	No answer	1.6%	0.9%	1%	2.8%
Religion	Roman Catholic	54%	54.6%	65.3%	43%
	Orthodox	0.63%	0%	1%	0.9%
	Buddhist	0.31%	0%	0%	0.9%
	Atheist	30.68%	25%	26.5%	40.2%
	Agnostic	6.39%	8.3%	3.1%	7.5%
	Native believer	2.24%	1.9%	0%	4.7%
	Other	1.6%	2.9%	0%	1.9%
	No answer	4.15%	7.4%	4.1%	0.9%
Place of residence	Rural area	19.2%	24.1%	15.3%	17.8%
	Small city with up to 50 k residents	18.5%	19.4%	18.4%	17.8%
	City 51–100 k	8%	13.9%	5.1%	4.7%
	City 101–200 k	22.4%	22.2%	42.9%	3.7%

**Table 2:** Results concerning attitudes toward euthanasia, assisted suicide and palliative care held by medical and Polish and classical philology students.

Item		Total	First- to Fourth-Year Medical Students	Fifth- to Sixth-Year Medical Students and Intern	First- to Fifth-Year Students of the Polish and Classical Philology	*p*-Value
		(*n* = 313)	(*n* = 108)	(*n* = 98)	(*n* = 107)	
Attitudes toward euthanasia	Yes	68.69%	65.75%	65.3%	74.8%	0.072
	No	14.69%	11.1%	17.35%	15.9%	
	Do not know	16.61%	23.15%%	17.35%	9.3%	
Attitudes toward assisted suicide	Yes	35.8%	38%	30.6%	38.3%	0.398
	No	24.3%	19.4%	30.6%	23.4%	
	Do not know	39.9%	42.6%	38.8%	38.3%	
Attitudes toward palliative care	Yes	71.88%	68.5%	89.8%	58.9%	<0.001
	No	14.05%	11.1%	5.1%	25.2%	
	Do not know	14.05%	20.4%	5.1%	15.9%	

**Table 3:** Knowledge of the definitions of palliative care, euthanasia and assisted suicide and differences between knowledge of the definitions and attitudes.

	Yes	No	Attitudes toward Euthanasia	Attitudes toward Assisted Suicide	Attitudes toward Palliative Care
	%	%	*p*-Value	*p*-Value	*p*-Value
Knowledge of the definition of palliative care (*n* = 313)	87.5	12.5	0.972	0.533	0.463
Knowledge of the definition of euthanasia (*n* = 313)	95.5	4.5	0.138	0.916	0.744
Knowledge of the definition of assisted suicide (*n* = 313)	57.8	42.2	0.992	<0.001	0.330

**Table 4:** Readiness (of medical students and trainee doctors) to perform euthanasia and assisted suicide and declared willingness to undergo euthanasia (all respondents) and differences between readiness to perform euthanasia and assisted suicide and declared willingness to undergo euthanasia and attitudes.

	Yes		No	Do Not Know	Attitudes toward Euthanasia	Attitudes toward Assisted Suicide	Attitudes toward Palliative Care
	%		%	%	*p*-Value	*p*-Value	*p*-Value
Readiness to perform euthanasia (*n* = 206)	43.7		17.5	38.8	<0.001	<0.001	0.016
Readiness to perform assisted suicide (*n* = 206)	25.7		22.8	51.5	<0.001	<0.001	0.015
	Natural death	Euthanasia	Assisted suicide	Persistent therapy			
	%	%	%	%			
Declared willingness to undergo euthanasia (*n* = 313)	41.5	42.8	9.6	6.1	<0.001	<0.001	0.035

**Table 5:** The most important value in the doctor–patient relationship. Differences between the most important value in the doctor–patient relationship and attitudes toward euthanasia, assisted suicide and palliative care.

	Ethical Norms According to Which One Should Always Respect the Patient’s Life, Even in an Advanced Stage of an Incurable Disease	Absolute Freedom of Choice for the Patient as well as the Possibility of Shortening the Life by the Doctor	Other	Attitudes toward Euthanasia	Attitudes toward Assisted Suicide	Attitudes toward Palliative Care
	%	%	%	*p*-Value	*p*-Value	*p*-Value
The most important value in the doctor–patient relationship (*n* = 313)	28.1	49.5	22.4	<0.001	<0.001	0.053

## Data Availability

Data are contained within the article and Supplementary Materials.

## Supplementary Materials

The following supporting information can be downloaded at: https://www.mdpi.com/article/10.3390/healthcare12080833/s1, File S1: A study questionnaire.
