# Supplementary material for: Psychological Factors Influencing Attitudes towards Euthanasia, Assisted Suicide and Palliative Care among Medical Students and Doctors in Training
Source: Healthcare (Basel). 2024 Apr 15;12(8):833. doi: 10.3390/healthcare12080833 (PMC11050125; doi:10.3390/healthcare12080833)
Supplement: Supplementary file 1 [file healthcare-12-00833-s001.zip › healthcare-2914763-supplementary.pdf]

File S1. QUESTIONNAIRE

We kindly ask you to answer with honesty the questions below by circling one appropriate answer. The questionnaire is voluntary and anonymous. Thank you very much for completing the questionnaire.

Date:

Faculty:

Year of the studies:

Sex:

- € Female
- € Male
- € Other

Age:

Religious affiliation:

- € Atheist
- € Christian:
  - € Catholic
  - € Orthodox
  - € Protestant
- € Muslim
- € Buddhist
- € Other:

Place of permanent residence:

- € A village
- € A town of up to 50 000 people
- € A town of 50 000 – 100 000 people
- € A city of 100 000 – 200 000 people
- € A city of 200 000 – 500 000 people
- € A city larger than 500 000 people

1. Do you know the definition of palliative care?

a) Yes – please write the definition below
b) No

2. Can widespread access to palliative care reduce the number of requests terminally ill patients make for euthanasia?

a) Yes
b) No
c) I don’t know

3. To what degree have classes in palliative medicine helped you in preparing for care over terminally ill patients?

    a) Significant help
    b) Average help
    c) Insufficient help
    d) Not helpful at all – please write why and what improvements can be made

4. Do you think that proper palliative care provides the patient with a dignified life until the end?

    a) Yes
    b) No
    c) I don’t know

5. Do you know the definition of persistent therapy in medicine?

    a) Yes – please write the definition below
    b) No

6. Do you think that persistent therapy should be carried out in terminally ill patients?

    a) Yes
    b) No
    c) I don’t know

7. Do you know the definition of euthanasia?

    a) Yes – please write the definition below
    b) No

8. Do you know the definition of assisted suicide?

    a) Yes – please write the definition below
    b) No

9. Would you perform euthanasia if law allowed it?

    a) Yes
    b) No
    c) I don't know

10. Would you perform assisted suicide if law allowed it?

    a) Yes

b) No
c) I don’t know

11. In Poland, according to article 150, paragraph 1 of the penal code (statue passed on the 6 of June 1997): “One who kills a person on his demand or out of compassion for him is subject to incarceration for a period of three months to five years”; paragraph 2: In extraordinary cases the court may show exceptional leniency or even refrain from punishment” and article 151: “One who by persuasion or aid drives a person to attempt to take their own life is subject to incarceration for a period of three months to five years”

    In your opinion are the above quoted legal acts:
    a) Appropriate
    b) Insufficient protection from euthanasia and assisted suicide
    c) Too restrictive and should be remitted

12. Do you think that the legalization of euthanasia or assisted suicide could lead to abuse, i.e. use without the consent of patients, in the earlier stage of an incurable disease, or in patients with other diseases?

    a) Yes
    b) No
    c) I don’t know

13. Are you for the legalization of euthanasia?

    a) Yes
    b) No
    c) I don't know.

14. Are you for the legalization of assisted suicide?

    a) Yes
    b) No
    c) I don't know.

15. In your opinion, in the case of legalizing euthanasia, should the doctor's decision be legally supervised?

    a) Yes
    b) No
    c) I don't know.

16. Which values are most important to you when interacting with patients?

    a) Ethical norms according to which one should always respect the patients’ life, even in the advanced stage of terminal illness
    b) The patients absolute freedom of choice, including the pessibility to end his/her life with assistance from a doctor
    c) Other – please state which

17. If you were diagnosed with an incurable illness, would you want:

a) A natural death
b) Euthanasia
c) Assisted suicide
d) Persistent therapy

Please provide the reason for your decision:

18. Which problems would you consider especially difficult in your future contacts with terminally ill patients?

a) Treating pain and other symptoms
b) Psychological and spiritual problems
c) Breaking bad news to patients concerning a diagnosis and prognosis
d) Inability to cure an illness
e) Other – please state

19. Which problems, in your opinion, may be the reason for the request of terminally ill patients for euthanasia:

a) Pain and other physical symptoms
b) Mental and spiritual problems
c) There is no cure for the disease
d) Lack of sufficient medical care
e) Other - please state:

20. Do you have experience in caring for a seriously ill person?

a) Yes
b) No.

21. Have you ever accompanied anyone in dying:

a) Relative: Yes/No
b) Patient: Yes/No

22. Have you ever had or are you suffering from a serious or chronic disease?

a) Yes
b) No

Comments on the questionnaire:

Thank you very much for completing the survey!
